# Cardiac Metastasis from Poorly Differentiated Thyroid Carcinoma: A Rare Case Report and Review of the Literature

**DOI:** 10.32604/or.2026.079674

**Published:** 2026-07-16

**Authors:** Xin Qian, Xian Deng, Rongjia Zhang, Xu Li, Dehui Qiao, Xiaodong Chen, Hui Yang

**Affiliations:** Department of Thyroid Surgery, The Affiliated Hospital of Southwest Medical University, Luzhou, China

**Keywords:** Poorly differentiated thyroid carcinoma, cardiac metastasis, internal jugular vein tumor thrombus, tumor thrombus, case report

## Abstract

**Background:** Poorly differentiated thyroid carcinoma (PDTC) is a rare, aggressive malignancy. Cardiac metastasis from PDTC is exceedingly uncommon. We report early cardiac metastasis occurring shortly after radical thyroidectomy to highlight atypical distant spread and management challenges. **Case Description:** A 62-year-old woman presented four months after thyroidectomy with progressive exertional dyspnoea, fatigue, productive cough, facial oedema, lip cyanosis, and dizziness. Postoperative pathology showed poorly differentiated thyroid carcinoma of the right lobe and isthmus (pT2N1bM0) with capsular and recurrent laryngeal nerve invasion. Transthoracic echocardiography revealed a right atrial mass that enlarged to 5.3 × 4.0 cm and extended toward the tricuspid orifice; imaging also demonstrated continuous tumor thrombus involving the right internal jugular vein, brachiocephalic vein, and superior vena cava. Cytology from internal jugular vein puncture showed malignant cells, supporting metastatic intraluminal tumor thrombus with intracardiac extension. After surgery, levothyroxine was initiated as thyroid hormone replacement and for gradual thyroid-stimulating hormone (TSH) suppression as tolerated. During cardiology admission, the patient received antimicrobial therapy, anticoagulation, thrombolysis, and cardiac workload reduction, with symptomatic improvement. She was discharged against medical advice before definitive oncologic treatment could be initiated and was subsequently lost to follow-up. **Conclusions:** In aggressive thyroid cancers such as PDTC, clinicians should remain vigilant for atypical distant metastases—including intracardiac extension via venous tumor thrombus—soon after surgery. Early multimodality imaging and coordinated multidisciplinary care are essential for individualized management.

## 1 Introduction

Thyroid cancer, the most common endocrine malignancy worldwide, encompasses a spectrum of histological subtypes with distinct biological behaviors and prognoses [1]. Among these, poorly differentiated thyroid carcinoma (PDTC) is a rare but clinically significant entity, comprising 2–13% of all thyroid cancers [2]. PDTC occupies an intermediate position between well-differentiated thyroid carcinomas (e.g., papillary and follicular) and anaplastic thyroid carcinoma, and is more invasive and carries a worse prognosis than well-differentiated tumors [3]. The standard definition of PDTC includes solid, trabecular or insular growth patterns and at least one high-grade feature—mitotic activity (>3 per 10 high-power fields), necrosis or nuclear irregularity. These features underlie its aggressive behaviour and propensity for local invasion and lymph-node metastasis, whereas distant metastasis is relatively uncommon [4].

Despite its relatively low incidence, PDTC presents substantial diagnostic and therapeutic challenges. Ultrasound-guided fine-needle aspiration cytology (FNAC) is commonly used to diagnose PDTC, but its sensitivity for this subtype is relatively low [5]. When preoperative cytology is non-diagnostic, additional biopsy or histopathological examination may be required. Management of PDTC primarily entails surgery, with total thyroidectomy and lymph-node dissection as the first-line approach. Complete surgical resection is a favorable prognostic factor [6,7]. Owing to the tumour’s aggressiveness, some patients have extrathyroidal extension or residual disease after surgery, necessitating adjuvant therapy such as radioiodine (RAI), external-beam radiotherapy (EBRT) or targeted therapy [8]. The role of RAI in PDTC remains controversial; some studies suggest benefit, whereas others show limited effects on survival [9]. EBRT is a feasible option for local disease control in selected cases, particularly those with large tumours, extrathyroidal extension, lymph-node metastasis or distant metastases [10]. Targeted therapies, including tyrosine-kinase inhibitors, show promise for advanced or metastatic PDTC, although their efficacy and optimal use remain under investigation [11,12].

Distant metastasis in PDTC is relatively uncommon; the lungs and bones are the most frequent sites [13]. Cardiac metastasis is exceptionally rare and potentially life-threatening. The mechanisms of cardiac metastasis in thyroid cancer are incompletely understood and may include haematogenous dissemination, lymphatic spread, direct invasion, or intraluminal extension via the superior and inferior venae cavae [14,15]. Given its rarity, data on clinical features, diagnostic strategies and treatment outcomes are limited. Management typically requires a multidisciplinary team—including endocrinology, oncology, surgery, radiology and cardiology—to individualize treatment and optimise outcomes.

We report cardiac metastasis from PDTC in a 62-year-old woman. We describe the clinical presentation, diagnostic work-up and treatment course, highlighting the challenges and considerations in managing this complex, rare condition. We also review the literature to contextualise current understanding of cardiac metastasis in PDTC and discuss implications for future research and clinical practice.

This study was approved by the Ethics Committee of The Affiliated Hospital of Southwest Medical University with the reference number: KY2026155. Written informed consent was obtained from the patient for participation in this case report. Additionally, specific written informed consent for publication of the clinical images and medical information was obtained from the patient, ensuring compliance with privacy and ethical standards. This report was prepared in accordance with the CARE case report guideline, and a CARE checklist was provided [16]. Please see Supplementary Material S1 for more details.

## 2 Case Report

This case report describes a 62-year-old woman who presented with a painful, mobile right-sided neck mass and hoarseness at the Affiliated Hospital of Southwest Medical University. She had no family history of cancer and had undergone pacemaker implantation 8 years earlier. On admission, ultrasound showed a 3.2 × 2.3 × 2.5 cm hypoechoic nodule in the right thyroid lobe (C-TIRADS 4B). Flexible fibreoptic laryngoscopy demonstrated right vocal-cord paralysis. Contrast-enhanced Computed Tomography (CT) of the neck suggested a right thyroid adenoma without additional abnormalities, while chest CT showed cardiomegaly related to prior pacemaker implantation. Ultrasound-guided FNAC of the thyroid nodule was Bethesda category VI, consistent with thyroid carcinoma. Following preoperative evaluation, the patient underwent extended radical resection of right thyroid carcinoma under general anaesthesia on 11 October 2024. Intraoperatively, the tumour invaded the right recurrent laryngeal nerve, necessitating partial resection. Postoperative pathology demonstrated poorly differentiated thyroid carcinoma (3.5 × 3.2 × 1.5 cm) involving the right lobe and isthmus, with capsular and recurrent laryngeal nerve invasion. Lymph node dissection revealed metastasis in 2 of 12 central nodes and 1 of 27 right-neck nodes. Immunohistochemistry showed CK19(+), TG(+), TTF-1(+), galectin-3(+), HBME-1(partial +), BRAF V600E (partial +), and Ki-67 (40%), with negative CD56, CgA, Syn, and CT. The pathological stage was pT2N1bM0. Postoperative adjuvant treatment options, including radioactive iodine, external beam radiotherapy, and targeted therapy evaluation, were discussed with the patient, but she declined these interventions and chose levothyroxine therapy with outpatient follow-up.

Postoperatively, levothyroxine was initiated at 75 μg/day primarily for thyroid hormone replacement, with subsequent TSH suppression planned to be titrated according to tolerance and follow-up thyroid function because of the patient’s underlying cardiac history. On 20 February 2025, she was admitted to cardiology with a one-month history of fatigue, exertional dyspnoea, productive cough, facial oedema, lip cyanosis, and dizziness. Transthoracic echocardiography revealed a 3.9 × 4.4 cm mildly echogenic mass attached to the right atrium with limited mobility, and heterogeneous low echogenicity in the superior vena cava and right internal jugular and subclavian veins with reduced cardiac pulsatility (Fig. 1). Follow-up echocardiography on 19 March 2025 showed enlargement of the right-atrial mass to 5.3 × 4.0 cm, with multiple hypoechoic areas and limited mobility extending to the tricuspid orifice (Fig. 2). Positron Emission Tomography/Computed Tomography (PET/CT) demonstrated low-attenuation filling defects in the right internal jugular, brachiocephalic and superior vena cava, consistent with tumor thrombus, and a high-attenuation lesion in the right atrium, possibly tumor thrombus (Fig. 3). Cytology from internal jugular-vein puncture confirmed malignant cells. During hospitalisation, she received antimicrobial therapy, anticoagulation, thrombolysis and measures to reduce cardiac workload, with improvement in dyspnoea and fatigue. She was discharged on 21 March 2025 after symptomatic improvement but requested discharge against medical advice before multidisciplinary reassessment of definitive oncologic treatment. She subsequently did not attend scheduled outpatient visits and was lost to follow-up (Table 1).

**Figure 1:**
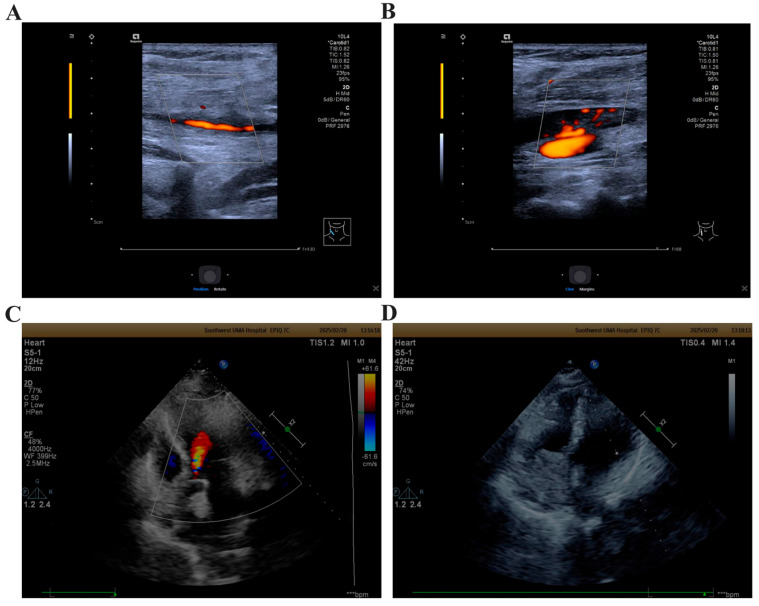
Neck vein and cardiac colour Doppler ultrasound on 20 February 2025. (**A**) Right internal jugular vein: heterogeneous hypoechoic intraluminal material with partial flow, suggestive of venous tumor thrombus. (**B**) Superior vena cava: extensive heterogeneous hypoechoic filling defects with reduced flow, consistent with venous tumor thrombus. (**C**) Transthoracic echocardiography (apical four-chamber view): 3.9 × 4.4 cm mildly echogenic right atrial mass with limited mobility and turbulent flow, consistent with intracardiac tumor thrombus. (**D**) Two-dimensional transthoracic echocardiography: right atrial mass with heterogeneous echotexture and multiple hypoechoic areas, extending toward the tricuspid orifice.

**Figure 2:**
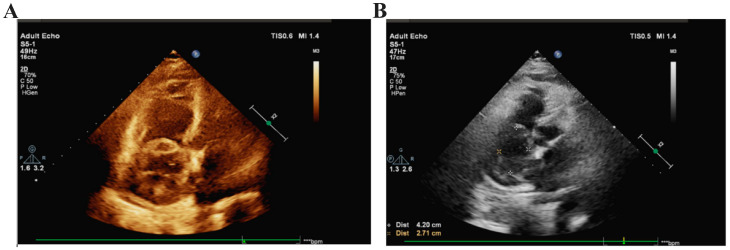
Transthoracic echocardiography on 19 March 2025. (**A**) Apical four-chamber view showing an enlarged right atrial mass with heterogeneous echotexture and multiple hypoechoic areas, indicating tumor thrombus progression. (**B**) Two-dimensional measurement view demonstrating the enlarged right atrial mass measuring 5.3 × 4.0 cm, with limited mobility and extension toward the tricuspid valve orifice.

**Figure 3:**
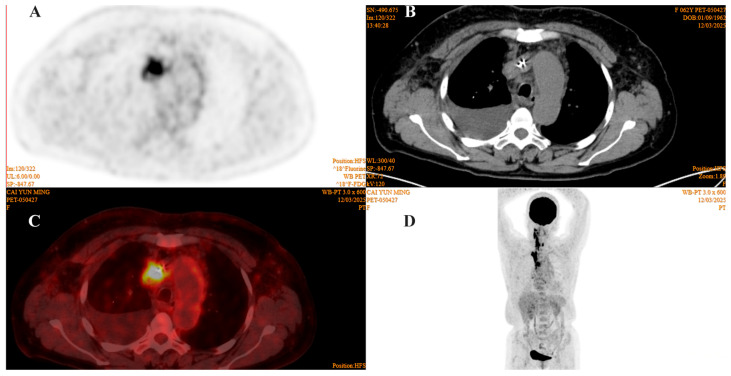
PET/CT on 12 March 2025, showing intraluminal tumor thrombus with intracardiac extension. (**A**) Axial PET image showing focal increased ^18^F-FDG uptake in the superior mediastinum corresponding to the right atrial mass and venous tumor thrombus. (**B**) Axial CT image demonstrating low-attenuation filling defects in the superior vena cava and right brachiocephalic vein, consistent with extensive venous tumor thrombus. (**C**) Axial fused PET/CT image showing intense FDG avidity within the right atrial mass and continuous venous tumor thrombus, confirming metabolically active neoplastic involvement. (**D**) Whole-body maximum intensity projection (MIP) image demonstrating the extent of disease with abnormal FDG uptake in the neck and superior mediastinum, corresponding to the venous tumor thrombus and intracardiac extension.

**Table 1:** Timeline of clinical events.

Date/Timepoint	Key Symptoms/Signs	Diagnostic Evaluation (Key Tests & Results)	Interventions/Treatment	Outcome/Follow-Up
≈2017	—(Past history)	Permanent pacemaker implantation.	—	—
2024-10-11	Right-sided neck mass, hoarseness.	Surgery & postoperative pathology (simplified): PDTC of the right lobe/isthmus (3.5 × 3.2 × 1.5 cm) with capsular and right RLN invasion; nodal metastases 2/12 central, 1/27 right lateral; stage pT2N1bM0. IHC supportive: CK19, TG, TTF-1, galectin-3 positive; HBME-1 and BRAF V600E focal positive; CD56 negative; Ki-67 hot spot ≈ 40%.	Extended right thyroid cancer resection with partial resection of the invaded right RLN under general anesthesia.	Uncomplicated postoperative recovery.
2024-10 to 2025-01	—	—	Levothyroxine 75 μg daily was initiated for thyroid hormone replacement, with planned gradual TSH suppression according to tolerance and thyroid function.	Routine postoperative follow-up.
≈2025-01 onward	Progressive exertional dyspnea and fatigue, with productive cough, facial edema, lip cyanosis, dizziness (suggestive of SVC syndrome).	—	—	Symptoms progressively worsened.
2025-02-20 (cardiology admission)	As above.	TTE: right atrial (RA) hypo-echoic mass ~3.9 × 4.4 cm with restricted mobility; heterogeneous hypoechoic material in SVC/right IJV/subclavian vein; reduced cardiac pulsatility.	Inpatient multidisciplinary management.	Symptomatic care initiated.
2025-03 (during hospitalization)	—	PET/CT: continuous filling defects in the right IJV, brachiocephalic vein, and SVC, with associated right atrial involvement, overall favoring intraluminal tumor thrombus rather than bland thrombus.	Antimicrobial therapy, anticoagulation, thrombolysis, preload reduction, and supportive care.	Symptoms gradually improved.
2025-03-19	—	Repeat TTE: RA mass increased to 5.3 × 4.0 cm with multiple hypo-echoic areas, extending toward the tricuspid valve orifice; restricted mobility.	Continued inpatient therapy and reassessment.	—
2025-03 (later in stay)	—	Cytology from right IJV puncture: malignant cells → consistent with cardiac metastasis with tumor thrombus.	Multidisciplinary reassessment of definitive oncologic therapy was planned after clinical stabilization; however, this could not be completed because the patient requested discharge against medical advice.	Diagnosis established.
2025-03-21 (discharge)	Dyspnea and fatigue improved vs. baseline.	—	Discharged after symptomatic improvement; continued levothyroxine; definitive oncologic treatment deferred pending reassessment.	Last confirmed contact at discharge; subsequent outpatient visits were missed and the patient was lost to follow-up.

Note: PDTC, Poorly differentiated thyroid carcinoma; RLN, Recurrent laryngeal nerve; LN, Lymph node; IHC, Immunohistochemistry; CK19, Cytokeratin 19; TG, Thyroglobulin; TTF-1, Thyroid transcription factor-1; HBME-1, Hector battifora mesothelial-1; Ki-67, Proliferation index (hot spot); SVC, Superior vena cava; IJV, Internal jugular vein; RA, Right atrium; TTE, Transthoracic echocardiography; PET/CT, Positron emission tomography/computed tomography; MDT, Multidisciplinary team; TSH, Thyroid-stimulating hormone; AJCC, American joint committee on cancer (8th ed. staging). ‘—’ indicates that no relevant information, procedures, examinations, symptoms, or specific content were documented for that dimension.

## 3 Discussion

PDTC is a rare subtype of thyroid carcinoma, comprising 2–13% of all thyroid malignancies. It occupies an intermediate position between well-differentiated thyroid carcinomas (e.g., papillary and follicular) and anaplastic thyroid carcinoma (ATC). PDTC is highly invasive and carries a poor prognosis, with a reported 10-year survival of 66.5–79.9% [17]. We report cardiac metastasis from PDTC, a rare manifestation that further complicates management and worsens prognosis.

PDTC typically presents with local invasiveness and a propensity for lymph-node metastasis, whereas distant metastasis is less frequent [18]. This patient exhibited several high-risk features, including age ≥ 55 years, invasion of the thyroid capsule and recurrent laryngeal nerve, and nodal metastasis at diagnosis. These features are associated with increased risk of recurrence and a poor prognosis. Diagnosis is often challenging because ultrasound-guided FNAC has relatively low sensitivity for PDTC. When preoperative cytology is non-diagnostic, additional biopsy or histopathological examination may be required.

Cardiac metastasis from thyroid cancer is exceptionally rare, with only a few cases reported. The mechanisms underlying cardiac metastasis are incompletely understood and may include haematogenous dissemination, lymphatic spread, direct invasion, or intraluminal extension via the superior and inferior venae cavae [4]. In this case, the patient developed intraluminal tumor thrombus in the internal jugular vein with subsequent cardiac metastasis, presenting with superior vena cava (SVC) syndrome and symptoms of increased cardiac workload. The cardiac lesion represented intraluminal tumor thrombus with venous extension rather than myocardial parenchymal deposit, as evidenced by echocardiographic demonstration of a mobile, mildly echogenic mass attached to the right atrium with heterogeneous echotexture, and PET/CT showing continuous low-attenuation filling defects from the internal jugular vein through the brachiocephalic vein and superior vena cava to the right atrium. The cardiac and venous tumor thrombus in this case represents hematogenous metastasis with intraluminal extension, occurring four months after complete surgical resection of the primary tumor, consistent with M1 disease rather than T4b direct invasion. Tumor cells likely invaded the venous wall, forming tumor thrombus that propagated to the heart via the superior vena cava.

Management of PDTC primarily entails surgery, with total thyroidectomy and lymph-node dissection as the first-line approach. Complete surgical resection is a favourable prognostic factor. Owing to the tumour’s aggressiveness, some patients have extrathyroidal extension or residual disease after surgery, necessitating adjuvant therapy such as RAI, EBRT or targeted therapy. The role of RAI in PDTC remains controversial; some studies suggest benefit, whereas others show limited effects on survival. EBRT is a feasible option for local disease control in selected cases, particularly those with large tumours, extrathyroidal extension, lymph-node metastasis or distant metastases [19].

In this case, levothyroxine was initiated after surgery primarily for thyroid hormone replacement, with gradual TSH suppression intended according to clinical tolerance. Although postoperative adjuvant treatment options were discussed, the patient declined further oncologic intervention after thyroidectomy. Following the subsequent development of venous tumor thrombus with intracardiac extension, she received multidisciplinary conservative management including anticoagulation, thrombolysis, antimicrobial therapy, and cardiac workload reduction, resulting in short-term symptomatic improvement. Management of cardiac metastasis in PDTC remains challenging because no standardised treatment pathway exists, and individualized decision-making is required according to tumor behavior, hemodynamic status, treatment tolerance, and patient preference.

Overall prognosis in PDTC is poor; cardiac metastasis further complicates management and increases mortality risk. Because cardiac metastasis is rare, data to guide clinical practice are limited. Future research should elucidate the molecular mechanisms that drive PDTC aggressiveness and metastasis. Priorities include defining genetic and epigenetic alterations that drive tumour progression and identifying actionable therapeutic targets. In addition, developing more effective diagnostic tools and standardised treatment protocols for cardiac metastasis in PDTC is essential to improving patient outcomes.

In this case, the patient’s age ≥ 55 years, invasion of the thyroid capsule and recurrent laryngeal nerve, and nodal metastatic involvement at the time of surgery contributed to a high risk of recurrence and a poor prognosis. Early development of internal jugular-vein tumor thrombus and cardiac metastasis underscores the need for vigilant surveillance for distant metastasis, particularly in high-risk patients. The management of intracardiac tumor thrombus posed significant therapeutic challenges. Although surgical resection or endovascular intervention could theoretically offer definitive treatment, these options were deferred at the time of admission due to the patient’s hemodynamic instability and poor functional status. Her presentation with exertional dyspnoea, facial oedema, lip cyanosis, and fatigue indicated limited tolerance for major invasive procedures. Consequently, we instituted an initial conservative strategy comprising anticoagulation, thrombolysis, antimicrobial therapy, and cardiac workload reduction to achieve clinical stabilization. We had planned to convene a multidisciplinary team discussion to evaluate the risk-benefit ratio of surgical or interventional approaches once her condition improved. However, the patient requested discharge against medical advice following symptomatic improvement, precluding completion of this evaluation and definitive intervention. This case highlights the importance of multidisciplinary care, with collaboration among endocrinology, oncology, surgery, radiology and cardiology, particularly given the absence of standardised treatment protocols for cardiac metastasis. To further contextualize the present case, we summarized representative patterns of cardiac involvement reported in thyroid carcinoma and compared them with the current patient (Table 2).

**Table 2:** Comparison of representative patterns of cardiac involvement among thyroid carcinoma subtypes and the present case.

Feature	Incidence of Cardiac Metastasis	Timing of Cardiac Metastasis	Mechanism	Presentation	Iodine Avidity	Treatment	Prognosis
PDTC (Present Case)	Extremely rare (<5 cases)	4 months post-surgery (short interval)	Venous tumor thrombus extension	Right heart failure, SVC syndrome	Likely reduced or uncertain iodine avidity	Conservative stabilization initially; definitive treatment not completed	Poor; aggressive
Follicular Thyroid Carcinoma	Rare (~20–30 cases)	3–10 years after diagnosis	Venous tumor thrombus extension (characteristic)	SVC syndrome, right atrial mass	Usually preserved	RAI + surgery	Moderate to good if resectable
Papillary Thyroid Carcinoma	Very rare (~10–15 cases)	5–20 years after diagnosis	Hematogenous to myocardium	Often asymptomatic, arrhythmia	Usually preserved	RAI + surgery	Variable; generally better
Anaplastic Thyroid Carcinoma	Rare but most common (~30–40 cases)	At presentation or within months	Direct invasion or hematogenous	Rapidly progressive, widespread disease	Lost	Chemotherapy/TKIs, poor response	Very poor; <6 months median survival

Note: PDTC, Poorly Differentiated Thyroid Carcinoma; SVC, Superior Vena Cava; RAI, Radioactive Iodine; TKI, Tyrosine Kinase Inhibitors; FNAC, Fine Needle Aspiration Cytology; IHC, Immunohistochemistry; CK19, Cytokeratin 19; TG, Thyroglobulin; TTF-1, Thyroid Transcription Factor-1; HBME-1, Hector Battifora Mesothelial-1; Ki-67, Proliferation Index (Hot Spot).

This case highlights several strengths, including multimodality imaging, cytological confirmation from internal jugular vein puncture, and detailed documentation of rapid intraluminal progression from the cervical veins to the right atrium. It also has important limitations. First, this is a single-case report and its findings cannot be generalized. Second, pathological confirmation was obtained from venous cytology rather than direct sampling of the intracardiac lesion. Third, dedicated multiplanar cardiac PET/CT reconstructions were unavailable because the examination was performed as a whole-body staging study. Fourth, the patient declined further invasive evaluation and was subsequently lost to follow-up, limiting assessment of long-term outcome and treatment efficacy. Despite these limitations, the case broadens recognition of an unusual metastatic pattern of PDTC and underscores the need for early multidisciplinary assessment in high-risk patients.

## Data Availability

The data supporting the findings of this study are available from the corresponding author upon reasonable request.

## Supplementary Materials

The supplementary material is available online at https://www.techscience.com/doi/10.32604/or.2026.079674/s1.

## Abbreviations

PDTC	poorly differentiated thyroid carcinoma
FNAC	fine-needle aspiration cytology
RAI	radioiodine
EBRT	external-beam radiotherapy
ATC	anaplastic thyroid carcinoma
SVC	superior vena cava
TSH	thyroid-stimulating hormone
