# Supplementary material for: Cardiac Metastasis from Poorly Differentiated Thyroid Carcinoma: A Rare Case Report and Review of the Literature
Source: Oncol Res. 2026 Jul 16;34(8):29. doi: 10.32604/or.2026.079674 (PMC13397374; doi:10.32604/or.2026.079674)
Supplement: Supplementary file 1 [file OncolRes-34-79674-s001.zip › TSP_OR_79674-Supplementary_Material_S1.docx]

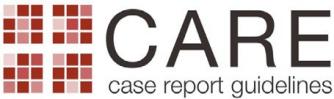

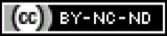

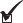
**CARE Checklist of information to include when writing a case report**

| **Topic** | **Item No** | **Checklist item description** | **Reported on Page Number/Line Number** | **Reported on Section/Paragraph** |
| --- | --- | --- | --- | --- |
| Title | 1 | The diagnosis or intervention of primary focus followed by the words “case report” | p. 1 | Title |
| Key Words | 2 | 2 to 5 key words that identify diagnoses or interventions in this case report, including "case report" | p. 2 | Abstract, KEYWORDS |
| Abstract  (Structured summary) | 3a | Background: state what is known and unknown; why the case report is unique and what it adds to existing literature. | p. 1 | Abstract, Background |
|  | 3b | Case Description: describe the patient’s demographic details, main symptoms, history, important clinical findings, the main diagnosis, interventions, outcomes and follow-ups. | p. 1-2 | Abstract, Case Description |
|  | 3c | Conclusions: summarize the main take-away lesson, clinical impact and potential implications. | p. 2 | Abstract, Conclusions |
| Introduction | 4 | One or two paragraphs summarizing why this case is unique **(may include references)** | p. 2-3 | 1 Introduction, paras. 1-3 |
| Patient Information | 5a | De-identified patient specific information | p. 3-4 | 2 Case Report, paras. 1-2 |
|  | 5b | Primary concerns and symptoms of the patient | p. 3-4 | 2 Case Report, paras. 1-2 |
|  | 5c | Medical, family, and psycho-social history including relevant genetic information | p. 3 | 2 Case Report, para. 1 |
|  | 5d | Relevant past interventions with outcomes | p. 3-4 | 2 Case Report, paras. 1-2 |
| Clinical Findings | 6 | Describe significant physical examination (PE) and important clinical findings | p. 3-4 | 2 Case Report, paras. 1-2 |
| Timeline | 7 | Historical and current information from this episode of care organized as a timeline | p. 7-9 | Table 1 (Timeline of clinical events) |
| Diagnostic Assessment | 8a | Diagnostic testing (such as PE, laboratory testing, imaging, surveys). | p. 3-9 | 2 Case Report, paras. 1-2; Figs. 1-3; Table 1 |
|  | 8b | Diagnostic challenges (such as access to testing, financial, or cultural) | p. 13 | Not explicitly reported; diagnostic limitations noted in 3 Discussion, final paragraph |
|  | 8c | Diagnosis (including other diagnoses considered) | p. 4, 8-9 | 2 Case Report, para. 2; Table 1 |
|  | 8d | Prognosis (such as staging in oncology) where applicable | p. 4, 10-11 | 2 Case Report, para. 1 (pT2N1bM0); 3 Discussion, paras. 1-2, 5-6 |
| Therapeutic Intervention | 9a | Types of therapeutic intervention (such as pharmacologic, surgical, preventive, self-care) | p. 3-4, 8-9 | 2 Case Report, paras. 1-2; Table 1 |
|  | 9b | Administration of therapeutic intervention (such as dosage, strength, duration) | p. 4 | 2 Case Report, para. 2 (levothyroxine 75 μg/day) |
|  | 9c | Changes in therapeutic intervention (with rationale) | p. 3-4, 8-9 | 2 Case Report, paras. 1-2; Table 1 |

| Follow-up and Outcomes | 10a | Clinician and patient-assessed outcomes (if available) | p. 4, 8-9 | 2 Case Report, para. 2; Table 1 |
| --- | --- | --- | --- | --- |
|  | 10b | Important follow-up diagnostic and other test results | p. 4, 7-9 | 2 Case Report, para. 2; Figs. 1-3; Table 1 |
|  | 10c | Intervention adherence and tolerability (How was this assessed?) | p. 3-4, 8-9 | 2 Case Report, paras. 1-2; Table 1 (patient declined adjuvant/definitive therapy) |
|  | 10d | Adverse and unanticipated events | p. 4, 8-9 | 2 Case Report, para. 2; Table 1 (rapid progression, discharge against medical advice, lost to follow-up) |
| Discussion | 11a | A scientific discussion of the strengths AND limitations associated with this case report | p. 13 | 3 Discussion, final paragraph |
|  | 11b | Discussion of the relevant medical literature **with references** | p. 2-3, 10-13 | 1 Introduction, paras. 1-3; 3 Discussion |
|  | 11c | The scientific rationale for any conclusions (including assessment of possible causes) | p. 10-13 | 3 Discussion, paras. 1-6 |
|  | 11d | The primary “take-away” lessons of this case report (without references) in a one paragraph conclusion | p. 2, 11-13 | Abstract, Conclusions; 3 Discussion, paras. 5-6 and final paragraph |
| Patient Perspective | 12 | The patient should share their perspective in one to two paragraphs on the treatment(s) they received | Not reported | Not reported in the current manuscript |
| Informed Consent | 13 | Did the patient give informed consent? Please provide if requested | Yes (p. 3, 14) | 1 Introduction, final paragraph; 5 Statements, Ethics Approval |

*As the checklist was provided upon initial submission, the page number/line number reported may be changed due to copyediting and may not be referable in the published version. In this case, the section/paragraph may be used as an alternative reference.
